# Using iPSC‐derived human DA neurons from opioid‐dependent subjects to study dopamine dynamics

**DOI:** 10.1002/brb3.491

**Published:** 2016-05-25

**Authors:** Yang Sheng, Emily Filichia, Elizabeth Shick, Kenzie L. Preston, Karran A. Phillips, Leslie Cooperman, Zhicheng Lin, Paul Tesar, Barry Hoffer, Yu Luo

**Affiliations:** ^1^ Department of Neurological SurgeryCase Western Reserve UniversityClevelandOhio44106; ^2^ Department of GeneticsCase Western Reserve UniversityClevelandOhio44106; ^3^ National Institute on Drug AbuseIntramural Research ProgramBaltimoreMaryland21224; ^4^ Department of PsychiatryMclean HospitalHarvard UniversityBelmontMassachusetts02478

**Keywords:** 3′ VNTR, hDAT gene, iPSCs, opioid dependent

## Abstract

**Introduction:**

The dopaminergic (DA) system plays important roles in addiction. However, human DA neurons from drug‐dependent subjects were not available for study until recent development in inducible pluripotent stem cells (iPSCs) technology.

**Methods:**

In this study, we produced DA neurons differentiated using iPSCs derived from opioid‐dependent and control subjects carrying different 3′ VNTR (variable number tandem repeat) polymorphism in the human dopamine transporter (*DAT* or *SLC6A3*). In addition, the effects of valproic acid (VPA) exposures on iPSC‐derived human DA neurons are also examined.

**Results:**

We present the first evidence suggesting that the 3′ VNTR polymorphism in the *hDAT* gene affects DAT expression level in iPSC‐derived human DA neurons. In human DA neurons, which provide an appropriate cellular milieu, VPA treatment alters the expression of several genes important for dopaminergic neuron function including *DAT, Nurr1, and TH;* this might partly explain its action in regulating addictive behaviors. VPA treatment also significantly increased DA D2 receptor (*Drd2*) expression, especially in the opioid‐dependent iPSC cell lines.

**Conclusions:**

Our data suggest that human iPSC‐derived DA neurons may be useful in in vitro experimental model to examine the effects of genetic variation in gene regulation, to examine the underlying mechanisms in neurological disorders including drug addiction, and to serve as a platform for therapeutic development.

## Introduction

Midbrain dopaminergic (DA) neurons in the ventral tegmental area (VTA) represent a common substrate for drugs of abuse and mediate the engagement of addictive behaviors (Wise 2008). There is strong evidence that the dopaminergic system that projects from the VTA of the midbrain to the nucleus accumbens (NAc) and to other forebrain sites, including the dorsal striatum and the prefrontal cortex, is the major substrate of reward and reinforcement for both natural rewards and addictive drugs (Wise et al. 1995; Hyman and Malenka 2001). Although drugs of abuse are chemically divergent molecules with very different initial activities, one of the common downstream actions is the activation of the mesolimbic dopamine system. This includes increased firing of dopamine neurons in the VTA and a subsequent increase in synaptic dopamine concentration in target brain areas including the NAc and prefrontal cortex (Nestler 2001). Pharmacological antagonists and lesion studies have also demonstrated that this dopaminergic pathway is required for reward‐related behaviors (Wise and Bozarth 1987; Koob and Bloom 1988). It would be of great importance to be able to directly assay the molecular and cellular mechanisms that regulate the mesolimbic dopamine system in individual patients.

Given the prominent role of dopamine as a neurotransmitter in natural and drug‐related reward pathways, genes that are involved in the neurotransmitter actions of dopamine such as the dopamine transporter (*DAT* or *SLC6A3*) and the vesicular monoamine transporter 2 (*VMAT2* or *SLC18A2*) have been investigated for their potential role in drug abuse. Besides their convergent role for multiple addiction substances, DAT and VMAT2 are also direct targets of several drugs, such as cocaine and amphetamines (AMPH) (Shimada et al. 1991; Hyman et al. 2006; Krasnova and Cadet 2009). Rodent studies have demonstrated that DAT and VMAT2 play important roles in reinforcing behaviors (Hall et al. 2003; Thomsen et al. 2009).

Human DAT (*hDAT*) genes carry high densities of DNA polymorphisms. One of the most well‐studied polymorphisms in the human *DAT* gene is the variable number tandem repeats (VNTR) located in the 3′ untranslated region (3′ UTR) in exon 15 (3′ VNTR of 40 bp) (Shumay et al. 2010). This polymorphism in the *hDAT* gene has been consistently associated with cessation of smoking, obesity in smokers, ADHD, schizophrenia, and alcoholism (Heinz and Goldman 2000). Imaging studies have suggested that this genetic variation might affect the availability of DAT in the striatum of human subjects (van Dyck et al. 2005). Overexpression of variant hDAT constructs in cell lines suggests that the 9‐ or 10‐repeat VNTR can regulate dopamine transporter density (VanNess et al. 2005), but such studies are limited since they lack the proper cellular milieu. To understand how these candidate genes might contribute to the molecular mechanisms of drug abuse, it requires functional and specific cell types that carry the polymorphisms in candidate genes, originating from both drug‐dependent subjects and control subjects. Until recently, studying neurons carrying the genomic information from specific patients in the field of drug addiction has yet to be explored and past studies have been limited to postmortem tissue, blood samples, or imaging protocols.

To fully understand the mechanisms through which genetic variants affect vulnerability to drug abuse, the effects of such polymorphisms on the expression and function of encoded proteins need to be elucidated in individual patients. Recent developments in the induction of pluripotent stem cells from somatic adult cells provide a tremendous opportunity for this objective. The iPS cell technology enables the derivation of patient‐specific pluripotent stem cells which are a scalable platform for the in vitro differentiation into specific cell types of interest (Takahashi and Yamanaka 2006; Takahashi et al. 2007). Standardized protocols for derivation and differentiation of iPSCs from defined genetic backgrounds and phenotypes into specific cell classes in individuals provide an opportunity to study the cellular and molecular mechanisms of addiction. These cells would also provide useful tools for screening potential therapeutic compounds for the treatment of addiction and toxicity of drugs. iPS cell technologies have been used to examine the mechanisms of multiple disorders including amyotrophic lateral sclerosis (ALS) (Egawa et al. 2012), Huntington's disease (HD) (HD iPSC Consortium 2012), and Parkinson's disease (PD) (Devine et al. 2011). However, to the best of our knowledge, only one pilot study reported research using human iPSC‐derived neural cells in alcohol abuse (Lieberman et al. 2012) and no study has been reported using human iPSC‐derived dopaminergic neurons in addiction.

In this article, we present the first evidence suggesting that the 3′ VNTR polymorphism affects human DAT expression level in iPSC‐derived human dopaminergic neurons. In addition, we further evaluate the effects of valproic acid exposure on iPSC‐derived human DA neurons.

## Materials and Methods

### Participants inclusion criteria and buccal swabs collection

Inclusion criteria for all participants aged 21–65 years old were as follows: for the opioid‐dependent group only, enrollment in a substance abuse treatment protocol at the NIDA (National Institute of Drug Abuse) Intramural Research Program; for the nondrug users, no lifetime history of drug dependence as indicated by the screening Addiction Severity Index (McLellan et al. 1985) and Substance Abuse/Dependence Evaluation counselor interview. Exclusion criteria included: (1) relevant neurological disorders (including, but not limited to, Parkinson's disease and Huntington's disease); (2) contraindications to skin biopsy including, but not limited to, bleeding disorders, skin disorders, and immune disorders, that the Medical Advisory Investigator (MAI) determines may alter the risk of the biopsy; (3) cognitive impairment severe enough to preclude informed consent or valid responses on questionnaires; (4) nondrug users were also excluded if they tested positive for drugs or alcohol during screening or study visits; (5) unwillingness to allow samples to be kept for future research. This study was reviewed and approved by the NIH Addictions Institutional Review Board. Participants gave prior written informed consent and were paid for completing the research components of the study.

Buccal swabs were used to collect cells for genetic characterization. Participants were asked not to eat or drink anything for at least 30 min before the procedure. A cotton swab was rubbed firmly against the inside of each cheek several times. The swab was labeled with a code and then stored until shipped to Case Western University for genetic testing. Based on the results of the DNA testing of *DAT* polymorphisms and balanced between drug use histories (opioid‐dependent and control), participants were asked to return for a second study visit for collection of a skin biopsy. A urine specimen for drug screening was also collected.

### Genotyping of DNA samples from opioid‐dependent and control participants

Genomic DNA samples, obtained from buccal swabs, were extracted using the Qiagen DNA mini kit (cat # 51304). Genomic DNA from each individual was subjected to PCR to type the 3′ VNTR polymorphism for *hDAT*. All the genotyping was carried out as previously described (Vandenbergh et al. 1992). PCR amplification was performed using the primers (DATVNTRF: 5′‐TGTGGTGTAGGGAACGGCCTGAG‐3′ and DATVNTRR: 5′‐CTTCCTGGAGGTCACGGCTCAAGG‐3′) as reported previously (Vandenbergh et al. 1992).

We enrolled and completed buccal swabs on 22 opioid‐dependent participants (12 African American, 7 White, 1 Asian, and 1 more than one race) and 27 control participants (20 African American, 6 White, and 1 more than one race). Based on the genetic findings, we completed skin biopsies on 10 opioid‐dependent and 10 control participants from whom fibroblast cell lines were developed on five opioid‐dependent and six control participants. For this study, we completed the derivation of four iPSC lines from fibroblasts (each of the genotypes 10/10 or 9/9 from control and opioid‐dependent subjects). hiPSCs were generated by reprogramming the fibroblasts derived from control and opioid‐dependent subjects that carry either 9 or 10 repeats of the *hDAT* 3′ VNTR (details shown in Table 1).

**Table 1:** Summary of the subjects from which iPS cells are derived

Participant	Group	Allele	Ethnicity	Gender
1	Opioid‐dependent	10/10	African American	Male
2	Opioid‐dependent	9/9	White	Male
3	Control	10/10	More than one race	Male
4	Control	9/9	White	Female

### Isolation and characterization of skin fibroblasts from drug addicts and controls

A full‐thickness punch biopsy of the skin was obtained from selected genotyped participants as described earlier. Biopsy samples were cultured in DMEM supplemented with 10% fetal bovine serum to allow fibroblasts to grow out from the biopsy. Fibroblasts were passaged twice and cryopreserved for future iPSCs generation.

### Transcription factor‐mediated reprogramming of fibroblasts into iPSCs

Fibroblasts were reprogrammed into iPSCs using standard techniques as described previously (Guo et al. 2013). In brief, VSV‐g pseudo‐typed lentivirus containing a polycistronic coding sequence for the human genes *OCT3/4, SOX2, KLF4, and MYC* (hSTEMCCA vector kindly provided by Dr. Gustavo Mostoslavsky, Boston University School of Medicine) was used to infect fibroblasts. Seven days after infection, fibroblasts were transferred to a cell feeder layer of mouse embryonic fibroblasts and fed every other day with human pluripotent stem cell media consisting of DMEM/F12 (Invitrogen, Carlsbad, CA) supplemented with 20% knockout serum replacement (KSR; Invitrogen), 2 mM glutamax (Invitrogen), 1× nonessential amino acids (Invitrogen), and 10 ng/mL FGF2. At day 21 after infection, individual iPSC clones were manually picked and dissociated for expansion into iPSC lines.

### Dopaminergic differentiation of iPSC cell lines

iPSCs were differentiated into dopaminergic cells using the floor‐plate‐based strategy as previously described (Kriks et al. 2011). Briefly, midbrain floor‐plate precursors were derived from iPSCs without feeder cells after 11 days of exposure to small molecule activators of sonic hedgehog (SHH) and canonical WNT signaling. We derived midbrain floor‐plate precursors from hiPSCs 11 days after exposure to combined shh/Pur/FGF8 and dual SMAD inhibition (exposure to LDN193189 + SB431542) in addition to CHIR99021 (CHIR), a known strong activator of WNT signaling. The precursor cells were then further matured into dopaminergic neurons in neurobasal/B27 medium supplemented with ascorbic acid, BDNF, GDNF, TGF*β*3, cAMP, and DAPT as described previously (Kriks et al. 2011). Cells were passaged onto poly‐l‐ornithine/laminin/fibronectin‐coated plates on days in vitro (DIV) 18 and cultured in the neuronal differentiation media until DIV28 before analyzed for various assays.

### Valproic acid treatment

Valproic acid was obtained from Sigma Aldrich. DA neurons were treated with 0.6 mM of valproic acid starting on DIV23 to DIV28 for 5 days before being harvested for various assays.

### Immunocytochemistry

DA neuronal cultures were immunostained with antibodies against various dopaminergic neuronal markers as described below. DA neuronal cultures were fixed with 4% paraformaldehyde in PBS for 15 min at room temperature (RT) and washed with PBS and permeabilized with 0.1% Triton‐X in PBS for 5 min before blocking for 1 h in 10% goat or donkey serum in PBS. Cells were then incubated with primary antibodies: TH (Rabbit polyclonal, Millipore 1:1000, Billerica, MA), TUJ‐1 (Mouse monoclonal, Millipore, 1:1000 Billerica, MA), VMAT2 (Rabbit polyclonal, Millipore, 1:1000, Billerica, MA), Nurr1 (Goat polyclonal, R&D, 1:500 Minneapolis, MN) diluted in 10% serum in PBS at 4°C overnight. After three washes in PBS, the cells were incubated with fluorescent secondary antibodies at RT for 1 h. Cells were counterstained with DAPI and images were acquired using an Olympus microscope (Olympus, Center Valley, PA). Omission of the primary or secondary antibodies resulted in no staining and served as negative controls. Percentage of TH‐positive cells and percentage of TUJ‐1‐positive cells were quantified using Nikon NIS‐Elements software and averaged from 10 randomly selected fields in each culture well. The percentage was determined in 6–9 wells of cells for each line pooled from three independent experiments and the SEM refers to the standard errors among these 6–9 wells for each line.

### Quantitative reverse transcription‐PCR (qRT‐PCR)

Dopaminergic cultures were harvested at DIV28 for total RNA extraction following instructions from the manufacturer (RNAqueous, Ambion). Total RNA (1 *μ*g) was treated with RQ‐1 Rnase‐free Dnase I and reverse transcribed into cDNA using the Superscript III reverse transcriptase kit. cDNA levels for *GAPDH, HPRT1, Nurr1, TH, DAT, VMAT2, Drd2, ptx3, mu, kappa, and delta opioid receptors* were determined by specific universal probe library primer probe sets (Roche) using Roche Light Cycler II 480. Primers and FAM‐labeled probes used in the quantitative RT‐PCR for each gene are listed in Table 2. Relative expression level was calculated using ΔΔCt method compared to *GAPDH* as a reference gene. All of the results were obtained with triplicate samples for each line/conditions/experiment and were combined from at least two independent experiments.

**Table 2:** Primer/probe sets used in qRT‐PCR

Primer/probe set	
GAPDH	
Forward primer	5′‐AGC CAC ATC GCT CAG ACA C‐3′
Reverse primer	5′‐GCC CAA TAC GAC CAA ATC C‐3′
Probe	Universal probe library: Probe 60 – Roche
HPRT1	
Forward primer	5′‐TGA TAGATC CAT TCC TAT GAC TGT AG‐3′
Reverse primer	5′‐AAG ACA TTC TTT CCA GTT AAA GTT GAG‐3′
Probe	Universal probe library: Probe 22 – Roche
TH	
Forward primer	5′‐GCC AAG GAC AAG CTC AGG‐3′
Reverse primer	5′‐AGC GTG TAC GGG TCG AAC T‐3′
Probe	Mouse universal probe library: Probe 42 – Roche
DAT	
Forward primer	5′‐CAA CAA GTT CAC CAA CTG C‐3′
Reverse primer	5′‐GGA GGA GAA GCT CGT CAG G‐3′
Probe	Universal probe library: Probe 10 – Roche
VMAT2	
Forward primer	5′‐CGG GAT TCT GCA TCA TGT TT‐3′
Reverse primer	5′‐TGG CAA TCA GCA GGA AGG‐3′
Probe	Universal probe library: Probe 67 – Roche
Drd1	
Forward primer	5′‐TTG AGA GAG ACG ACC CCA AG‐3′
Reverse primer	5′‐TGT CTT CTC GCT CCT CCA A‐3′
Probe	Universal probe library: Probe 73 – Roche
Drd2	
Forward primer	5′‐TGA ACA GGC GGA GAG TGG‐3′
Reverse primer	5′‐GCT GGT GCT GGA GAG CAT‐3′
Probe	Universal probe library: Probe 17 – Roche
Ptx3	
Forward primer	5′‐CAG CAG CTA CAG GAG CTA GAG G‐3′
Reverse primer	5′‐GCC GGT TCT TGA ACC ACA‐3′
Probe	Universal probe library: Probe 6 – Roche
Nurr1	
Forward primer	5′‐TGA AGA GAG ACG CGG AGA AC‐3′
Reverse primer	5′‐AAA GCA ATG GGG AGT CCA G‐3′
Probe	Universal probe library: Probe 63 – Roche
FOXA2	
Forward primer	5′‐CCC AAT CTT GAC ACG GTG A‐3′
Reverse primer	5′‐AAA TAA AGC ACG CAG AAA CCA‐3′
Probe	Universal probe library: Probe 85 – Roche
LMX1A	
Forward primer	5′‐TGG AGG AGA ACT TCC AAA GC‐3′
Reverse primer	5′‐CAG ACA GAC TTG GGG CTC AC‐3′
Probe	Universal probe library: Probe 3 – Roche
OCT4	
Forward primer	5′‐CTT CGC AAG CCC TCA TTT C‐3′
Reverse primer	5′‐GAG AAG GCG AAA TCC GAA G‐3′
Probe	Universal probe library: Probe 60 – Roche
PAX6	
forward primer	5′‐GGT TGG TAT CCG GGG ACT T‐3′
Reverse primer	5′‐TCC GTT GGA ACT GAT GGA GT‐3′
Probe	Universal probe library: Probe 46 – Roche
NKX2.2	
Forward primer	5′‐CGA GGG CCT TCA GTA CTC C‐3′
Reverse primer	5′‐GGG GAC TTG GAG CTT GAG T‐3′
Probe	Universal probe library: Probe 71 – Roche
KOR	
Forward primer	5′‐ACC CTT GAA GGC AAA GAT CA‐3′
Reverse primer	5′‐TGC AAG GAG CAC TCA ATG AC‐3′
Probe	Universal probe library: Probe 66 – Roche
MOR	
Forward primer	5′‐ACA GGC AAG GTG AGT GAT GTT‐3′
Reverse primer	5′‐CAC CAA CAT ATC AGG CTG TGA‐3′
Probe	Universal probe library: Probe 12 – Roche
DOR	
Forward primer	5′‐ATC ACC GCG CTC TAC TCG‐3′
Reverse primer	5′‐GGT GGC CGT CTT CAT CTT AG‐3′
Probe	Universal probe library: Probe 3 – Roche

GAPDH, Glyceraldehyde‐3‐Phosphate Dehydrogenase; HPRT1, Hypoxanthine Phosphoribosyltransferase 1; TH, tyrosine hydroxylase; DAT, dopamine transporter; VMAT2, vesicular monoamine transporter 2; Drd1, Dopamine Receptor D1; Drd2, Dopamine Receptor D2; Ptx3, Pentraxin 3; Nurr1, Nuclear receptor related 1 protein; FOXA2, Forkhead Box A2; LMX1A, LIM Homeobox Transcription Factor 1, Alpha; OCT4, octamer‐binding transcription factor 4; PAX6, Paired box protein 6; NKX2.2, NK2 Homeobox 2; KOR, kappa opioid receptor; MOR, mu opioid receptor; DOR, delta opioid receptor.

### HPLC measurements of DA and metabolites

DA neuronal differentiation in all iPSC cell lines were carried out as described earlier. On DIV28, culture media was removed and replaced with artificial cerebrospinal fluid (CSF) with KCl (60 mM) and incubated for 10 min. The aCSF containing KCl was then mixed with 1/10 volume of 1N PCA, frozen on dry ice and stored at −80° until analyzed for DA content using HPLC. Dopamine concentration was measured by high‐performance liquid chromatography (HPLC) as described previously (Smith et al. 2005).

### Statistics

Statistical analysis was performed using one‐ or two‐way analysis of variance (ANOVA) as appropriate, with Student–Newman–Keuls post hoc tests. *P*s < 0.05 were considered statistically significant.

## Results

### Generation of midbrain DA neurons and comparison of opioid‐dependent and control subjects with both 9/9 and 10/10 hDAT 3′ VNTR

**Figure 1:**
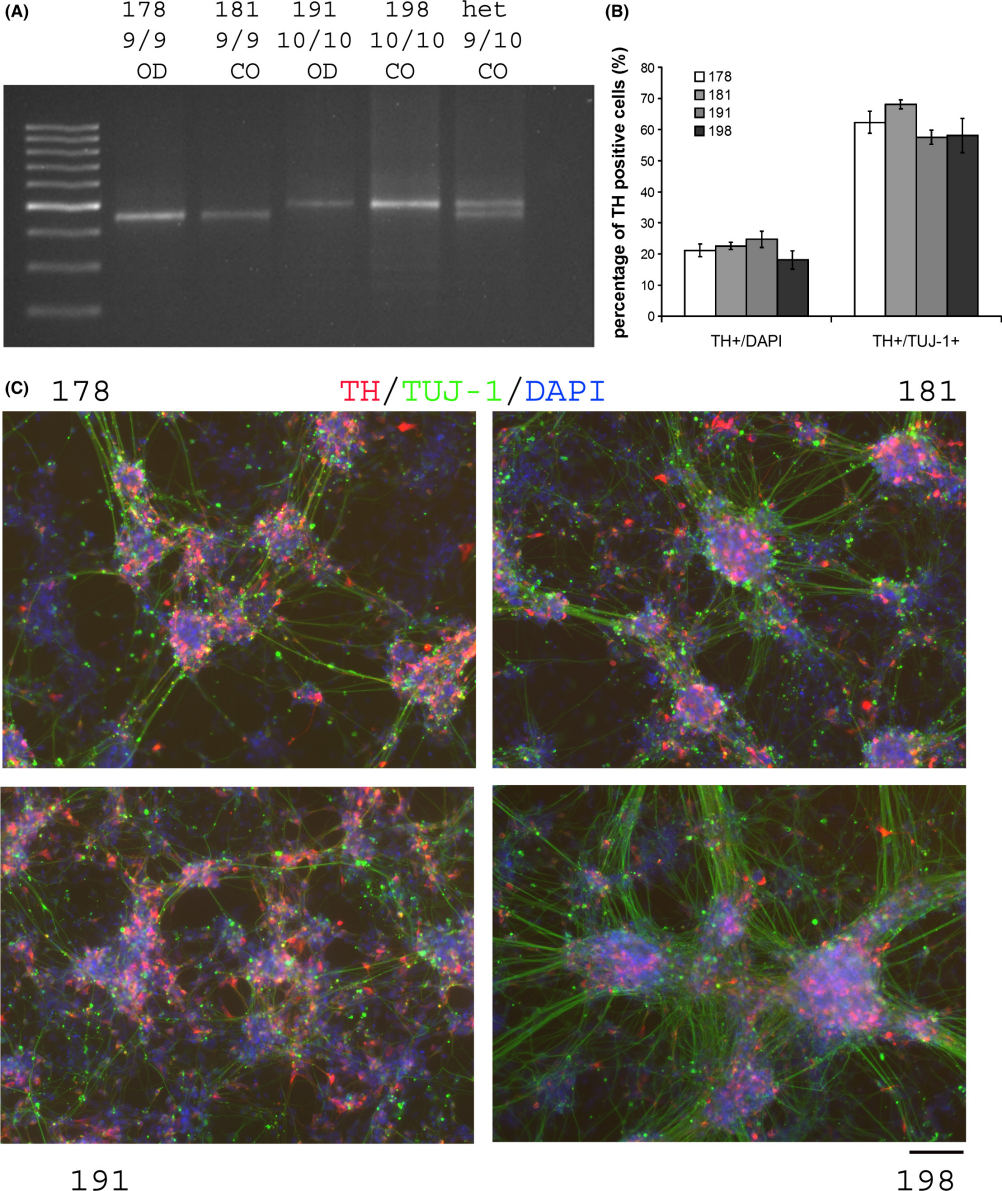
(A) Genotyping for hDAT 3′ VNTR repeat in iPSCs lines and (B) percentage of TH/TuJ‐1‐positive cells in DA differentiation culture in four cell lines. Percentage is calculated for 6–9 individual wells pooled from three independent experiments. Data are presented as mean ± SEM, SEM reflects the standard errors among the 6–9 individual wells for each cell line. (C) Representative images of differentiated DA neurons stained for TH/TUJ‐1/DAPI for all four cell lines. Scale bar = 100 *μ*m. OD, opioid dependent; CO, control.

Human dopaminergic neurons were differentiated from all four iPSC cell lines utilizing a recently developed novel floor‐plate‐based strategy (Kriks et al. 2011) as detailed above. DA neurons differentiated from this protocol express FOXA2, LMX1A, TH (tyrosine hydroxylase), Nurr1, VMAT2, and DAT (Sheng et al. 2016). We successfully generated “midbrain DA neurons” from all four iPSC lines derived in this study and we also utilized immunocytochemical double labeling to further validate the molecular identity of DA neurons. There was no apparent difference in DA differentiation efficiency between the cell lines derived from control and opioid‐dependent subjects (Fig. 1A–C) both with regard to the percentage of TH‐positive cells (21.19 ± 1.99%, 22.63 ± 1.16%, 24.70 ± 2.56%, 18.14 ± 2.94%, respectively, *F*(1, 29) = 0.00186, *P* = 0.966 for genotype and *F*(1, 29) = 1.645, *P* = 0.212 for control vs. opioid‐dependent, ANOVA), and the percentage of TH‐positive neurons with respect to TUJ‐1‐positive neuronal cells (62.26 ± 3.50%, 68.12 ± 1.48%, 57.48 ± 2.29%, 58.16 ± 5.44%, *F*(1, 29) = 1.757, *P* = 0.197 for genotype and *F*(1, 29) = 0.170, *P* = 0.844 for control vs. opioid‐dependent, ANOVA).

**Figure 2:**
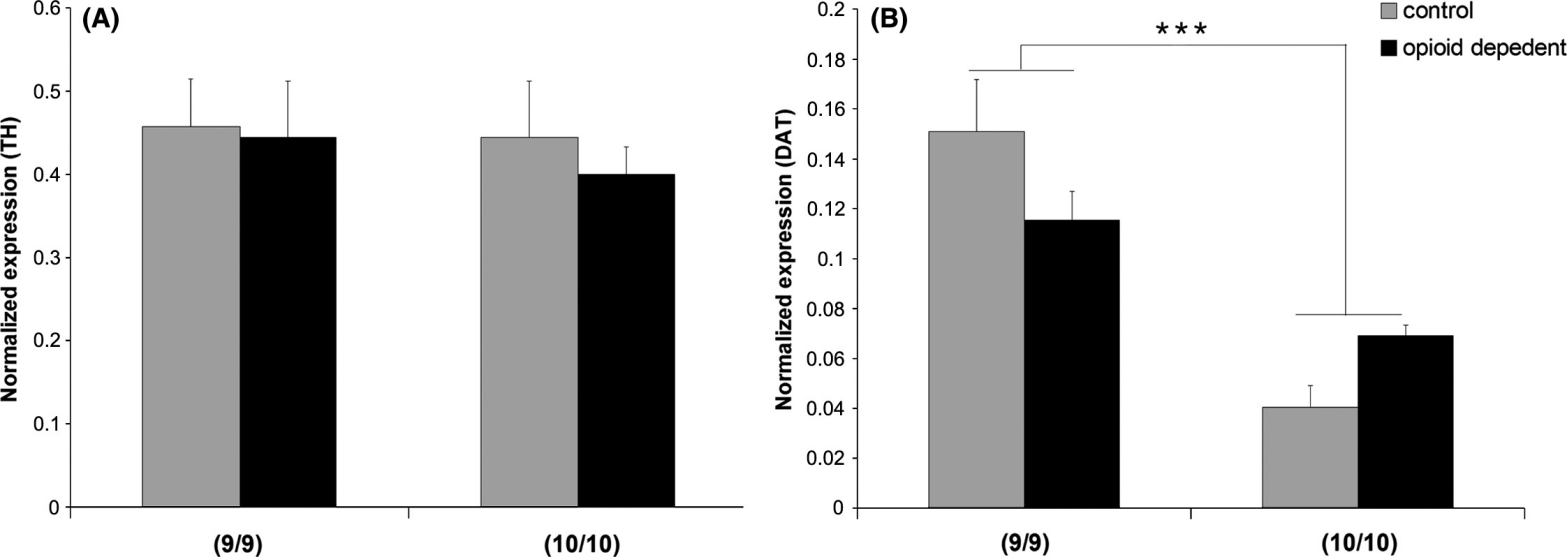
TH (A) and DAT (B) gene expression measured by qRT‐PCR in iPSCs‐derived DA neurons in different lines. At least six samples were harvested for RNA from each cell lines pooled from 2 to 3 independent experiments. No difference was observed in TH expression levels among different groups. *** indicates *P* < 0.001 for DAT levels 9/9 versus 10/10, ANOVA. No significant difference between control versus opioid‐dependent cells for DAT expression.

We next examined expression of the *DAT* gene in hDA neurons differentiated from iPSC lines that carry either 9 or 10 repeats of the hDAT 3′ VNTR. Our results showed that the TH expression level was equivalent among all the iPSC lines (Fig. 2A, *F*(1, 23) = 0.235, *P* = 0.633 for genotype and *F*(1, 23) = 0.228, *P* = 0.638 for control vs. opioid‐dependent, ANOVA). This is in agreement with the consistent TH‐positive % cells in different cell lines noted above. Interestingly, the 9/9 homozygous lines (control and opioid‐dependent) showed higher levels of DAT mRNA levels compared to the 10/10 lines (control and opioid‐dependent, Fig. 2B, *F*(1, 23) = 36.99, *P* < 0.001 for genotype, ANOVA) and there was no significant difference on DAT mRNA levels between control lines and opioid‐dependent lines (Fig. 2B, *F*(1, 29) = 0.0694, *P* = 0.795 for control vs. opioid‐dependent, ANOVA).

### Effects of valproic acid

**Figure 3:**
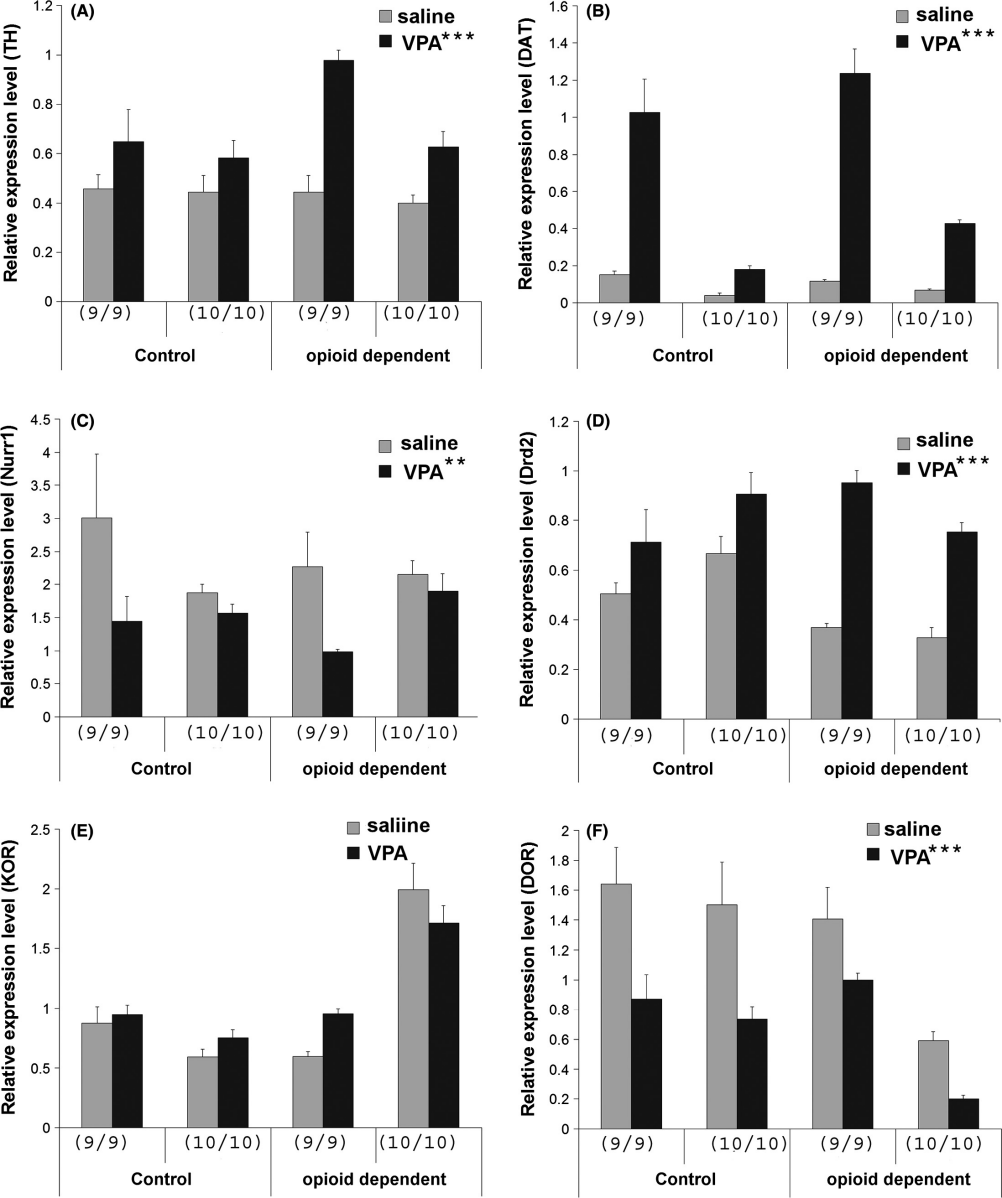
(A–F) Gene expression measured by qRT‐PCR in iPSC‐derived DA neurons in different lines treated with saline or 0.6 mM VPA from DIV23–28. At least six samples were harvested for RNA from each cell lines pooled from 2 to 3 independent experiments (*** or ** next to the VPA indicates *P* < 0.001 and *P* < 0.01 comparing saline versus VPA‐treated samples by ANOVA, # next to opioid‐dependent in panel D indicates difference in Drd2 expression in control versus opioid‐dependent lines for saline‐treated groups, *P* < 0.05, ANOVA). Ordinates in A–F indicate expression levels of each specific gene.

Recently, several lines of evidence suggest that valproic acid (VPA), a drug used in the treatment of mania and bipolar disorders, epilepsy, and addictions, may modulate dopamine transporter (DAT) function (Wang et al. 2007; Tsai et al. 2010; Chiu et al. 2011). We also thus tested whether our differentiated DA neurons respond to VPA treatment in a similar pattern as reported for primary rodent DA neurons from midbrain (Smith et al. 2010). Indeed, VPA treatment (0.6 mM for 5 days) substantially enhanced DAT transcription in all of the iPS cell lines (Fig. 3B, *F*(1, 47) = 36.118, *P* < 0.001, saline vs. VPA treatment, ANOVA). There was no difference in DAT mRNA levels between the control versus opioid‐dependent samples, *F*(1, 47) = 1.186, *P* = 0.282. Interestingly, VPA treatment also altered expression of other DA‐related genes in human DA neurons derived from our iPS cell lines. Specifically, TH and Drd2 receptors were both upregulated by VPA treatment in all four iPSC lines, (Fig. 3A, *F*(1, 47) = 24.370, *P* < 0.001 for TH expression, saline vs. VPA; Fig. 3D, *F*(1, 47) = 53.020, *P* < 0.001 for Drd2, saline vs. VPA, ANOVA), whereas the Nurr1 gene expression was downregulated by VPA treatment in all iPSC cells (Fig. 3C, *F*(1, 47) = 7.484, *P* < 0.01 for Nurr1 levels, saline vs. VPA, ANOVA). There was no difference in TH or Nurr1 expression levels between control and opioid‐dependent cell lines, (*F*(1, 47) = 2.053, *P* = 0.159, ANOVA for TH and *F*(1, 47) = 0.224, *P* = 0.639, ANOVA for Nurr1). We have reported recently that there are lower expression levels of Drd2 dopamine receptors in both of the opioid‐dependent iPSC lines compared to the DA neurons derived from control iPSCs (Sheng et al. 2016) which is consistent with previous neuroimaging findings showing that Drd2 receptor levels are lower in opioid‐dependent subjects (Volkow et al. 2004). Interestingly, although VPA treatment increased Drd2 expression in DA neurons derived from all four iPSC lines, the amount of increase in both of the opioid‐dependent lines were higher compared to both of the control iPSC lines (2.59‐ and 2.30‐fold of increases for opioid‐dependent lines vs. 1.42‐ and 1.35‐fold of increases in control iPSC lines). As a result, Drd2 levels are lower in opioid‐dependent lines in saline‐treated groups (*P* = 0.013, ANOVA, post hoc Student–Newman–Keuls Method) but not in VPA‐treated groups (*P* = 0.557, ANOVA, post hoc Student–Newman–Keuls Method), reflecting an interaction between cell origin and VPA treatment (*F*(1, 47) = 5.106, *P* = 0.029, two‐way ANOVA). Recently, we have also shown the expression of kappa and delta opioid receptor (KOR and DOR) but not mu opioid receptor (MOR) subtypes in human DA neurons differentiated from our iPSC cell lines (Sheng et al. 2016). MOR mRNA was not detected by qRT‐PCR using two independent primer/probe sets after 45 cycles of PCR amplification in our DA cultures from all four lines. No differences in KOR or DOR expression were found between control and opioid‐dependent lines (*F*(1, 47) = 0.0656, *P* = 0.799 for KOR and *F*(1, 47) = 1.671, *P* = 0.203 for DOR, ANOVA). VPA treatment did not result in consistent alteration of KOR receptor levels (Fig. 3E, *F*(1, 47) = 0.289, *P* = 0.594, saline vs. VPA, ANOVA) in our cell lines, but led to decreased DOR receptor levels in all cell lines (Fig. 3F, *F*(1, 47) = 16.571, *P* < 0.001, saline vs. VPA, ANOVA).

### DA neurochemistry

**Figure 4:**
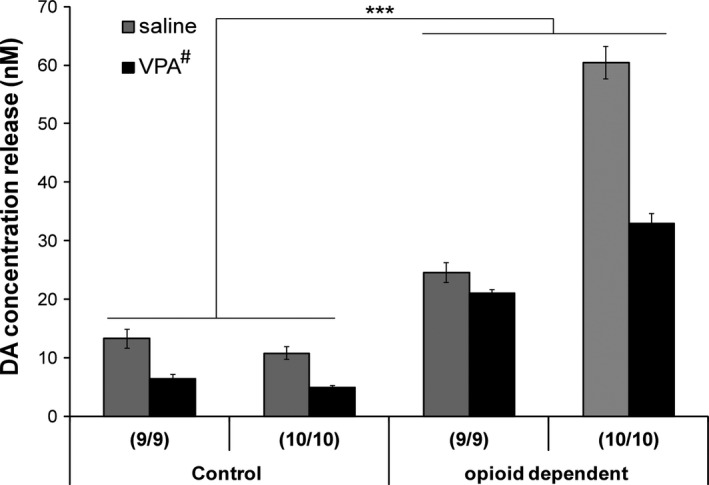
DA release measured by HPLC in various iPSC‐derived DA neurons treated with saline or 0.6 mM VPA from DIV23–28. *N* = 3 for each cell line/treatment, *** indicates *P* < 0.001, control versus opioid‐dependent lines, # next to VPA indicates *P* < 0.05, saline versus VPA treatment, ANOVA).

HPLC was used to examine DA release from our iPSC‐derived human DA neuronal cultures. Our data showed that in all of the iPSC cell line‐differentiated DA neurons, we were able to detect measurable amount of DA released using HPLC. DA neurons derived from opioid‐dependent subjects showed significantly higher levels of DA release compared to DA neurons derived from control subjects (Fig. 4, *F*(1,23) = 35.54, *P* < 0.001 for opioid‐dependent vs. control, ANOVA). Moreover, VPA treatment decreased DA release in DA neurons derived from all four lines (Fig. 4, average of 48.4%, 45.8%, 85.9%, and 54.6% of saline‐treated DA neurons, respectively, *F*(1, 23) = 6.296, *P* = 0.021, for saline vs. VPA, ANOVA).

## Discussion

This is the first molecular genetic characterization of iPSC‐derived DA neurons from drug opioid‐dependent versus controls. Although iPSCs have been used in multiple disease models including ALS, MS, and PD (Yamanaka 2009), the utilization of iPS cell technology for drug abuse has been limited. To the best of our knowledge, there has been only one pilot study that examined the response of iPSC‐derived neurons to alcohol (Lieberman et al. 2012). In this current study, our goal was to explore the potential utility of human iPSCs as a model to examine the effect of polymorphisms on gene expression comparing cells from opioid‐dependent versus control subjects and to examine the response of individually derived human dopaminergic neurons to addictive drugs and clinically relevant medications. To achieve this goal, we derived cell lines that carried polymorphisms that had previously been associated with drug addiction and compared cells lines that carried these polymorphisms derived from both control and opioid‐dependent subjects. Because our results were obtained only from control iPSCs and iPSCs derived from opioid‐dependent subjects, whether these findings apply to other types of drug addictions remain to be examined.

Utilizing the iPSC lines derived from well‐characterized subjects, we carried out a protocol that generates specifically FOXA2+/TH+ dopaminergic neurons using a recently developed FP‐based PSC differentiation protocol that faithfully recapitulates midbrain DA neuron development (Kriks et al. 2011). Indeed, our DA neuronal cultures expressed FOXA2, a unique property of the midbrain dopaminergic neurons as well as Nurr1, Ptx3, DAT, VMAT2, and the DA autoreceptor Drd2. In addition, we also detected DA release from our iPSC‐derived DA neurons, confirming that our cultures might provide a proper cellular model to be used in studies involving midbrain DA neurons in PD, schizophrenia, as well as in drug addiction.

Numerous clinical investigations have focused on the 3′ UTR VNTR in the *hDAT* gene (Shumay et al. 2010). This polymorphism in the *hDAT* gene has been consistently associated with cessation of smoking, obesity in smokers, ADHD, schizophrenia, and alcoholism (Heinz and Goldman 2000). However, the mechanism through which this, or other variants, in the *hDAT* gene might affect the expression and function of dopamine neurotransmission remains unknown. Overexpression studies have generated inconsistent and conflicting results (Fuke et al. 2001; Miller and Madras 2002; Mill et al. 2005; VanNess et al. 2005; van de Giessen et al. 2009), possibly due to the lack of correct cellular milieu and the “artificial” effects caused by a nonphysiological expression level. In this study, we derived iPSCs from individuals that carry different polymorphism variants (9/9 and 10/10 3′ UTR VNTR) from both control and drug abusers. Differentiating human iPSCs that carry this polymorphism enabled us to study functional alterations caused by certain genetic backgrounds in a more physiological cellular milieu. Our results showed that both of the 3′ UTR VNTR 9/9 lines (control and opioid‐dependent) have significantly higher levels of *DAT* transcripts compared to the two 3′ UTR VNTR 10/10 lines (control and opioid‐dependent), suggesting that this polymorphism does contribute to the regulation of *DAT* gene expression in human dopaminergic neurons derived from iPSCs. Previous studies using the overexpression analysis of reporter plasmids that contain either 9 or 10 repeat of VNTR generated inconstant results possibly due to the differences in the cell line used, in the construction of plasmids, and in the total amount of plasmid DNA transfected, all of which could confound the results. One previous study (Miller and Madras 2002) used two different promoters driving the luciferase reporter gene followed by the 9 or 10 repeat of 3′ UTR VNTR and carefully controlled the quality of plasmid DNA preparation and quantification. In that study, both of the two plasmid constructs showed that nine repeats of the VNTR resulted in a higher level of reporter expression which is consistent with our results obtained from human DA neurons. More importantly, a recent study using imaging techniques showed that human subjects that carry the 9/9 alleles have higher levels of striatal DAT expression compared to 10/10 alleles (van de Giessen et al. 2009). The fact that this human imaging study and our study found similar results regarding the effects of 9/9 or 10/10 alleles on the expression level of hDAT gene provides additional evidence that DA neurons differentiated from human iPSCs here might provide the proper cellular context for studying gene regulation and molecular mechanisms of DA neurotransmission.

Valproic acid (VPA), a branched short‐chain fatty acid, is widely used as an antiepileptic drug and a mood stabilizer (Chateauvieux et al. 2010). It has antiepileptic effects by inhibiting GABA neurotransmission and might be effective for the reduction of depressive symptoms in acute bipolar depression (Smith et al. 2010). In addition, VPA is neuroprotective in several models of neurodegenerative diseases (Monti et al. 2009, 2010; Chiu et al. 2011). VPA was also recently classified as a histone deacetylase inhibitor, carrying out its molecular action by inhibiting histone deacetylation which regulates gene transcription (Chateauvieux et al. 2010). It has been reported that VPA can regulate DAT expression in a dopaminergic cell line and rat midbrain DA neuronal cultures (Wang et al. 2007), however, whether VPA could regulate DAT expression in human DA neurons has not been examined previously. In this study, we tested the effect of VPA treatment on the expression of several key genes involved in DA neuron function. We selected a dose that has been previously demonstrated to be effective in rat DA neuronal cultures (0.6 mM) (Smith et al. 2010). Five days of low concentration VPA treatment dramatically increased DAT expression in all of the iPS cell lines we tested. Changes in DAT expression have been reported in multiple neurological disorders (reviewed in Bannon 2005). It has also been shown that DAT gene expression is significantly downregulated in long‐term cocaine users, which might be responsible for the increased likelihood of relapse (Bannon et al. 2004). A preclinical study has shown that multiple injections of VPA when delivered consecutively after methamphetamine could reduce the addictive behavior in rodents (Kalda et al. 2007). Consistently, clinical studies suggest that cocaine craving and abuse are diminished by treating cocaine abusers with valproate (Halikas et al. 2001; Zullino et al. 2004). These findings, taken together, suggest that VPA might be a good candidate for drug addiction modulation and treatment. Our data showed that in human DA neurons VPA treatment increases DAT expression, which might partly explain its action in regulating addictive behaviors. Interestingly, our data also showed that VPA treatment increased Drd2 receptor expression to a greater extent in the opioid‐dependent iPS cell lines, which exhibited lower expression levels in Drd2 receptors compared to control iPS cell lines in the absence of VPA. Thus, VPA treatment resulted in similar levels of Drd2 expression in both opioid‐dependent lines and control lines because VPA treatment preferentially enhanced Drd2 receptor expression in the opioid‐dependent lines. Since Drd2 receptor expression has consistently been shown to be reduced in a variety of addictive disorders (Volkow et al. 2007), this action of VPA treatment might provide another potential mechanism for its effect in addiction modulation and further supports its potential utility in drug addiction treatment.

DA release was also measured in our study and detectable levels of dopamine were released from our cultured dopaminergic neurons which further confirm that our DA cell models have an appropriate cellular and functional phenotype. DAT inhibitors were not used in this experiment because we wanted to measure the extracellular release of DA under physiological condition where no DAT inhibitors are present. Our data showed that VPA treatments resulted in a significant decrease of DA release in all four iPSC lines. This is consistent with the observation that VPA promotes DAT expression since increased DAT expression will lead to increased DA reuptake which in turn results in decreased DA release into the media. Serum VPA concentration has been studied previously; on average, a dose of 250 mg VPA induces a serum concentration of 54.6 *μ*g/mL (0.34 mM) (Hamilton et al. 1981), which is comparable to our tested concentration (0.6 mM) and this and higher doses are well tolerated (Atmaca et al. 2007). In summary, taken together, the data support the feasibility of testing VPA for future drug addiction treatment.

We also examined the expression of subtypes of opioid receptors in “DA neurons” differentiated from our iPS cell lines. Interestingly, our result showed that mu receptors are not detected in our human DA cultures by qRT‐PCR. These data support previous reports in animal studies that mu receptor are expressed in non‐DA neurons in midbrain (Garzon and Pickel 2001) and that mu receptor‐specific opioid agonists might excite DA neurons indirectly by modulating local non‐DA neurons such as GABA interneurons (Johnson and North 1992). In contrast, kappa opioid receptors have been detected in midbrain dopaminergic neurons in both mouse (Chefer et al. 2013) and human brain (Yamada et al. 1997; Peckys and Landwehrmeyer 1999) and kappa opioid agonists can directly inhibit midbrain dopaminergic neurons (Margolis et al. 2003). Delta opioid receptors have been reported to coexist with dopamine D1 receptors in striatal neurons (Ambrose et al. 2006). Whether delta opioid receptors are expressed in midbrain dopaminergic neurons has not been extensively examined; however, it was reported previously that stimulation of delta opioid receptors specifically in substantia nigra but not striatum restored motor activity in a PD animal model (Mabrouk et al. 2008) suggesting this opioid receptor subtype might play important roles in dopamine function in midbrain DA neurons. Interestingly, kappa and delta opioid receptors were both detected in our human DA neuronal cultures and VPA treatment has differential effects on them. VPA treatment did not lead to a significant alteration of KOR mRNA levels in our cell lines, but led to decreased DOR receptor levels in all cell lines. While DOR and KOR gene variants and expression have been associated with vulnerability to addiction (Margolis et al. 2008; Mendez and Morales‐Mulia 2008; Nielsen et al. 2013), some of the mechanisms involved were suggested to be acting through regulation of dopamine dynamics in vivo (Nielsen et al. 2013). Similarly, polymorphisms in *Drd2* gene and *DAT* VNTR polymorphisms can affect the efficacy and responses to methadone or buprenorphine maintenance treatment (Crettol et al. 2008; Gerra et al. 2014). Clearly there is an underlying interaction between the opioid receptors and dopamine transporter and receptors of which the exact molecular mechanisms need to be further examined in future studies.

In this study, we measured both dopaminergic‐specific genes (such as *TH, DAT, Nurr1, Drd2* as autoreceptor) as well as genes that can be expressed in other types of cells (such as *KOR and DOR*). Therefore, it is important to note that gene expression analysis was performed in a mixed population of cells. Technical advancement that allows for further purification of cultures for specific types of neurons would further improve the specificity of this type of analysis. In summary, our data obtained from this study suggest that human iPSC‐derived DA neurons may serve as a useful in vitro experimental model to examine the effects of genetic variation in gene regulation and to examine the underlying mechanisms in neurological disorders including drug addiction. In addition, iPSC‐derived DA neuronal cells may also provide a useful model to compare the differences between control and drug‐dependent subjects and to test toxicity and pharmacological responses to potential treatments on an individual basis.

## Conflict of Interest

None declared.
